# Double Aortic Arch: A Case Series and Multidisciplinary Surgical Experience

**DOI:** 10.1016/j.jaccas.2026.107057

**Published:** 2026-02-24

**Authors:** Jad Abdul Khalek, Karim Kanbar, Bshara Sleem, Rana Zareef, Issam El Rassi, Yehya Jassar, Fadi Bitar, Mariam Arabi

**Affiliations:** a Faculty of Medicine, American University of Beirut Medical Center, Beirut, Lebanon; b Division of Pediatric Cardiology, Department of Pediatric and Adolescent Medicine, American University of Beirut Medical Center, Beirut, Lebanon; c Division of Cardiothoracic Surgery, Department of Surgery, American University of Beirut Medical Center, Beirut, Lebanon

**Keywords:** aorta, computed tomography, congenital heart defect, imaging, pediatric surgery, postoperative, thoracotomy, treatment, ultrasound

## Abstract

**Background:**

Double aortic arch (DAA) is a rare congenital anomaly and the most common cause of a complete vascular ring. Although symptoms typically manifest in infancy, diagnosis may be delayed when clinical presentation is subtle or attributed to more common respiratory conditions.

**Case Summary:**

We report 3 patients with DAA who presented beyond infancy with long-standing, recurrent respiratory symptoms and feeding difficulties. In all cases, symptoms had been present for a prolonged period and were initially managed as recurrent respiratory or airway disease, resulting in delayed diagnosis. Imaging confirmed DAA with a right-dominant double aortic arch forming a complete vascular ring in each case. Surgical repair was performed via left thoracotomy, with posterior aortopexy required in 1 patient. All patients demonstrated marked postoperative clinical improvement.

**Discussion:**

Delayed presentation of DAA can occur when symptoms are nonspecific or intermittently progressive, leading to misdiagnosis or late referral. DAA should therefore be considered in children with persistent or recurrent respiratory or gastrointestinal symptoms unresponsive to standard therapy. Surgical correction via left thoracotomy is safe and effective, and results in substantial symptomatic relief with low morbidity.

**Take-Home Messages:**

DAA may present late due to nonspecific symptoms and diagnostic delay. Heightened clinical awareness, appropriate cross-sectional imaging, and timely surgical intervention are essential to prevent prolonged morbidity and achieve excellent outcomes.

## Visual Summary

**Figure:**
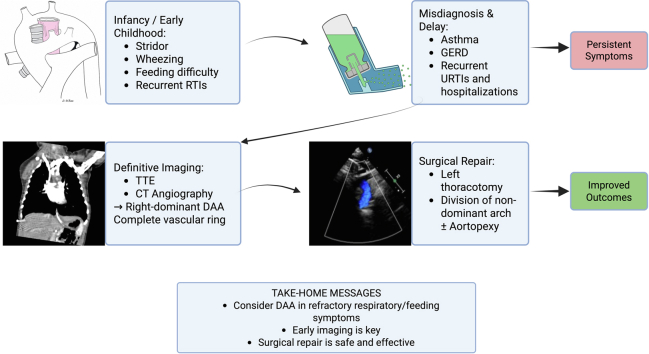

**Visual Summary:**
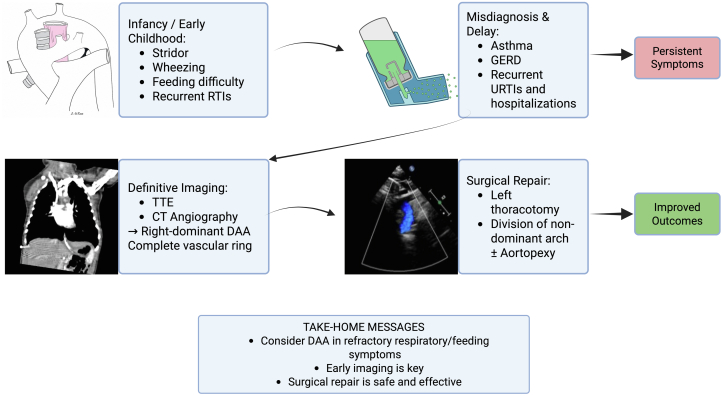
Diagnosis and Management of Double Aortic Arch Visual summary of the clinical presentation, diagnostic workflow, and management of double aortic arch, highlighting early respiratory and feeding symptoms that are often misdiagnosed, leading to delayed recognition. Definitive diagnosis with transthoracic echocardiography and CT angiography enables timely surgical repair, most commonly via division of the nondominant arch, resulting in improved clinical outcomes. CT = computed tomography; DAA = double aortic arch; GERD = gastroesophageal reflux disease; RTI = respiratory tract infections; TTE = transthoracic echocardiography; URTI = upper respiratory tract infections.

Double aortic arch (DAA) is the most common type of complete vascular ring, accounting for up to 55% of patients undergoing surgical correction for vascular rings. Although the exact incidence of DAA remains unknown, vascular rings as a group represent approximately 1% of congenital heart anomalies.^1^ There is no clear predilection for sex or ethnicity. DAA often presents in infancy or early childhood with symptoms of airway or esophageal compression, such as stridor, wheezing, or dysphagia.^2^ Associated congenital heart defects are reported in up to 12.6% of cases and may include ventricular septal defect, tetralogy of Fallot, or truncus arteriosus.^3^ Chromosomal abnormalities, including 22q11 deletion, trisomy 21, and trisomy 18, are also seen in up to 20% of cases.^1^^,^^4^^,^^5^ The incidence of DAA has increased with the routine use of first-trimester fetal ultrasound and is reported to be higher in pregnancies achieved via in vitro fertilization.^6^

**Take-Home Messages**

• A double aortic arch should be considered in infants and young children with persistent or recurrent respiratory or feeding symptoms, particularly when unresponsive to standard treatments.
• Early diagnosis requires high index of suspicion and prompt imaging, with echocardiography and CT angiography playing complementary roles.
• Surgical repair via left thoracotomy with division of the nondominant arch is safe and effective, offering excellent symptom relief and low complication rates.

**Figure 1:**
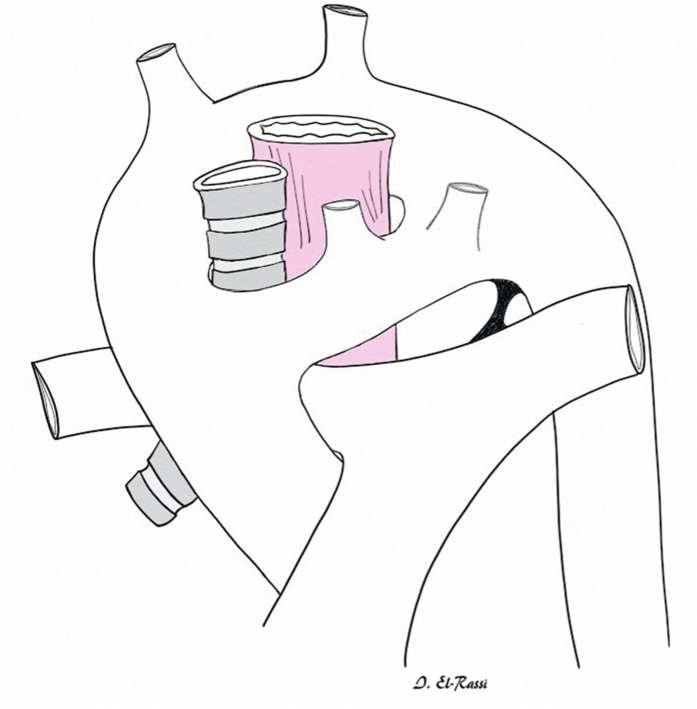
3-Dimensional Reconstruction Demonstrating a Complete Vascular Ring Due to a Double Aortic Arch

The development of the aortic arch is a complex process that takes place between the second and seventh weeks of embryonic life, whereby 6 pairs of arches form and then regress sequentially, leaving remnants that give rise to several major arteries in the mature circulatory system.^2^^,^^7^ Of importance is the fourth arch, which initially forms bilateral aortic arches, but by the fifth week of gestation, the right arch typically regresses while the left persists, ultimately resulting in a left-sided aortic arch.^2^^,^^7^ Thus, DAA results from the failure of regression of the right-sided arch alongside the persistence of the left-sided arch.^2^^,^^7^ This configuration forms a ring around the trachea and esophagus (Figure 1) and represents the most common cause of a complete vascular ring.^7^

Herein, we retrospectively describe case series of 3 patients diagnosed with DAA at the American University of Beirut Medical Center. Their presentation, medical history, imaging details, surgical intervention, and outcomes are all subsequently detailed.

## Results

### Case 1

**Figure 2:**
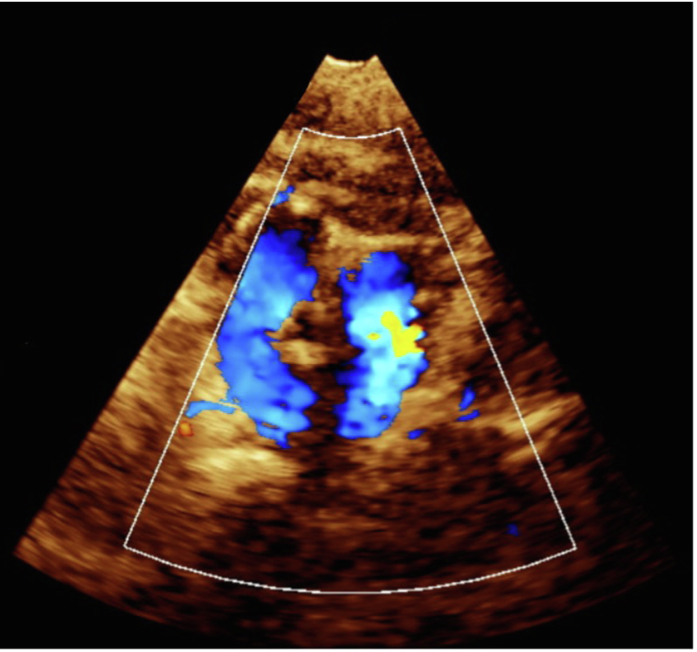
Transthoracic Echocardiography: Suprasternal Short-Axis View With Color Doppler, Demonstrating 2 Aortic Arch Limbs Encircling the Tracheal Air Column

**Figure 3:**
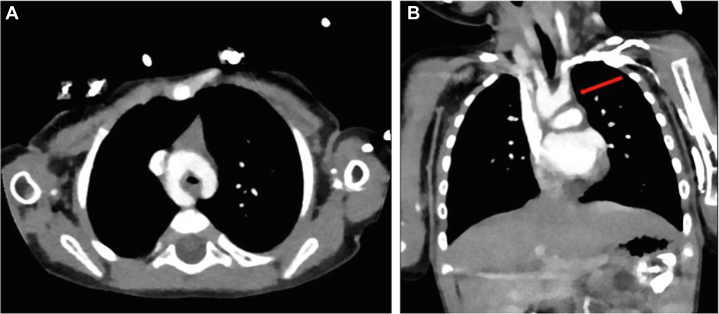
Computed Tomography Angiography Showing a Double Aortic Arch Forming a Complete Vascular Ring (A) Transverse view, and (B) coronal view highlighting the double arch (red arrow).

**Figure 4:**
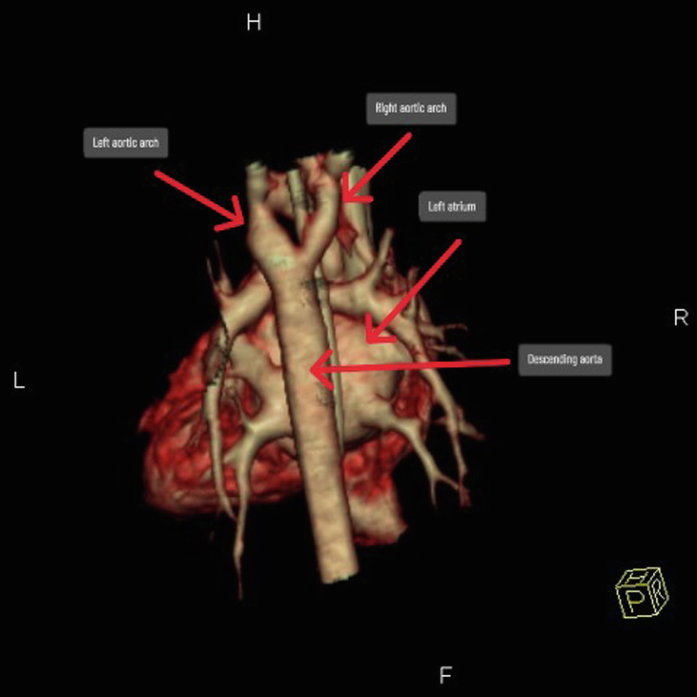
3-Dimensional Computed Tomography Angiography Reconstruction Showing a Double Aortic Arch Forming a Complete Vascular Ring

**Figure 5:**
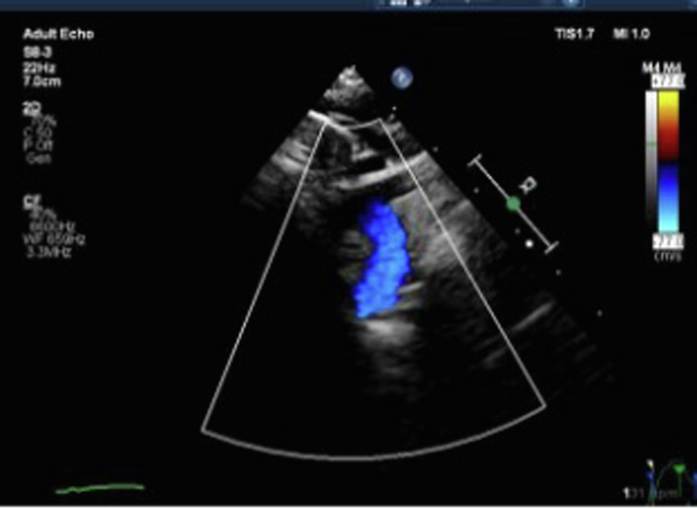
Follow-Up Transthoracic Echocardiography, Suprasternal View, Demonstrating the Aortic Arch 1 Month After Surgical Repair (Patient 1)

An 8-month-old girl presented to the pediatric cardiology clinic with a history of failure to thrive, recurrent vomiting, recurrent hospitalization, and intensive care unit admissions for respiratory distress. Previous investigations were performed at another institution, which included a barium swallow study, which revealed a suspicious area of esophageal narrowing; however, a final diagnosis was not reached. On physical examination, she had low weight for age (5.3 kg), tachypnea, subcostal retractions, stridor at rest, and severe secretions. Transthoracic echocardiogram (TTE) confirmed the diagnosis of DAA without associated intracardiac lesions (Figure 2, Video 1). This was followed by computed tomography angiography of the chest, which revealed a right dominant DAA and confirmed a complete vascular ring (Figures 3A, 3B and 4). She was admitted for surgical repair and was hospitalized for 6 days. Surgical correction was performed via a left thoracotomy, with division of the left aortic arch. The postoperative course was uneventful. TTE demonstrated pulsatile flow across the aortic arch, without an increase in velocity. At the 1-month follow-up visit, the patient exhibited mild respiratory symptoms, had normal feeding, and appropriate weight gain. Echocardiography (Figure 5) continued to demonstrate normal flow across the remaining arch, confirming a favorable surgical outcome. Notably, the patient is an orphan with no known family history, and additional preoperative findings included an absent or ectopically located left kidney.

### Case 2

**Figure 6:**
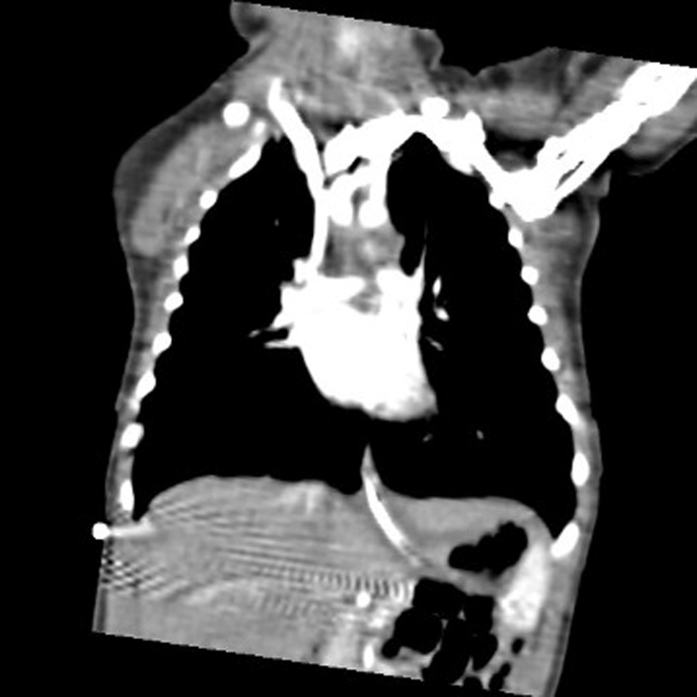
Computed Tomography Angiography of the Chest, Sagittal View, Confirming the Presence of a Double Aortic Arch

**Figure 7:**
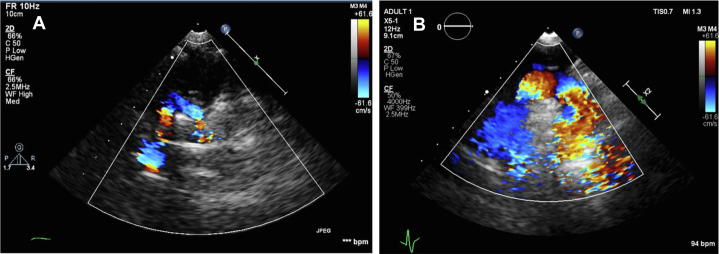
Transthoracic Echocardiography Comparison (A) Preoperative Doppler imaging demonstrating a right-dominant double aortic arch. (B) Postoperative suprasternal long-axis view with color Doppler showing unobstructed flow through the ascending aorta, aortic arch, and descending aorta.

A 3-year-and-2-month-old boy was referred to the pediatric cardiology clinic with a 6-month history of asthma, recurrent respiratory infections, chronic cough, wheezing, and intermittent episodes resembling breath-holding spells associated with perioral cyanosis. Chest radiography revealed right-sided tracheal compression with mild luminal narrowing. Otherwise, the patient exhibited normal growth and development. On physical examination, he had normal growth parameters (16 kg, 99 cm), without syndromic features. Family history was negative for congenital heart disease. TTE demonstrated a DAA, with right-dominant arch forming a complete vascular ring, with otherwise normal cardiac anatomy and function. Computed tomography angiography confirmed the diagnosis (Figure 6). The patient underwent surgical correction via left thoracotomy with transection of the left arch. Postoperative recovery was uneventful. Follow-up TTE revealed pulsatile flow across the aortic arch and descending aorta without evidence of increased velocity or residual obstruction. A comparison of preintervention and postintervention echocardiographic findings is shown in Figure 7.

### Case 3

A 21-month-old boy presented with recurrent respiratory infections requiring multiple hospital admissions since infancy, for which an extensive workup had been performed. He was treated for presumed gastroesophageal reflux and hyperactive airway disease without improvement and continued to experience episodes of choking and difficulty swallowing. History is significant for chronic benign neutropenia, for which the patient is maintained on granulocyte colony-stimulating factor.

TTE revealed a right-dominant DAA forming a complete vascular ring. The patient underwent surgical repair via left thoracotomy with division of the left aortic arch and posterior aortopexy, anchoring the aorta to the vertebral column distal to the left subclavian artery to relieve airway compression. Postoperatively, no complications were reported; feeding and respiratory symptoms improved. At 2-week follow-up, the patient was clinically stable with no respiratory distress, normal heart sounds, and clear lungs. At 2-month follow-up, he showed overall improvement postsurgery, with no choking or wheezing; however, mild noisy breathing and occasional upper respiratory tract infection persisted. Table 1 represents the pertinent information from the 3 cases.

**Table 1:** Patient Characteristics

	Patient 1	Patient 2	Patient 3
Age	8 months	3 years, 2 months	21 months
Sex	Female	Male	Male
Weight (kg)	5.3	16	11.4
Height (cm)	61	99	83
Blood pressure (mm Hg)	101/60	94/61	106/63
SpO_2_ (%)	97	100	100
Presenting symptoms	Stridor, recurrent vomiting, increased secretions, respiratory distress	Persistent cough, wheezing, and cyanotic episodes.	Recurrent respiratory infections and poor feeding
History	Recurrent ICU admissions due to respiratory distress	Asthma, recurrent URTI	Chronic benign neutropenia on GCSF, recurrent URTI
Initial imaging	Barium swallow: esophageal narrowing	Radiograph: mild narrowing of the right tracheal border	Radiograph: normal except for mild bilateral peribronchial thickening
Diagnostic imaging	TTE and CTA: codominant double aortic arch with complete vascular ring	TTE and CTA: right-dominant double aortic arch with complete vascular ring	TTE: right-dominant double aortic arch with complete vascular ring
Arch dominance	Right dominant	Right dominant	Right dominant
Surgical approach	Left thoracotomy	Left thoracotomy	Left thoracotomy
Procedure	Division of left aortic arch	Division of left aortic arch	Division of left aortic arch
Hospital stay (d)	6	7	6
Follow-up	Asymptomatic, good weight gain, normal feeding, preserved cardiac function. mild stridor, and tachypnea	Doing well with improvement noted; cough and stridor are improving; good cardiac function without increased velocity across the surgical site	Stable at 2 wk with good cardiac function with good weight gain and improved feeding; stable at 2 mo with improving respiratory symptoms.
Associated intracardiac anomalies	None	None	None
Other congenital findings	Absent or ectopic left kidney	None	None
Family history	Orphan; unknown family history	No family history	No family history

CTA = computed tomography angiography; GCSF = granulocyte colony-stimulating factor; ICU = intensive care unit; TTE = transthoracic echocardiography; URTI = upper respiratory tract infection.

## Discussion

DAA often presents in infancy or early childhood with nonspecific respiratory or gastrointestinal symptoms, making diagnosis challenging. Common presentations include stridor, wheezing, chronic cough, recurrent respiratory infections, feeding difficulties, choking episodes, or failure to thrive.^8^ These symptoms result from tracheoesophageal compression caused by the vascular ring. In our series, all 3 patients had symptoms that initially mimicked more common pediatric conditions. Two patients were diagnosed with asthma or hyperactive airway disease, and 1 was treated for gastroesophageal reflux without improvement. Recurrent hospital admissions for respiratory distress were common, and 1 patient had a history of recurrent intensive care admissions. Another presented with breath-holding spells and cyanosis, which raised concerns for airway compromise. Physical examination may be unremarkable or reveal signs such as stridor, tachypnea, intercostal retractions, or feeding difficulties. Auscultation may reveal wheezing or transmitted upper airway sounds, often leading to a mistaken diagnosis of reactive airway disease. This variability in presentation underlines the importance of maintaining a high index of suspicion for DAA in infants and young children with persistent or recurrent respiratory symptoms that are unresponsive to standard treatment, particularly when associated with feeding issues.

In our series, 2 patients were initially managed as asthma or gastroesophageal reflux disease before imaging revealed the vascular anomaly. The diagnostic workup begins with a chest radiograph, which may show tracheal deviation or narrowing. A barium swallow can reveal esophageal compression and is commonly the first clue. Definitive diagnosis is established through echocardiography and contrast-enhanced computed tomography angiography, which delineate the arch anatomy and confirm tracheoesophageal compression.^9^ Differential diagnoses include asthma, laryngomalacia, tracheomalacia, gastroesophageal reflux disease, or other congenital airway lesions.^1^ Early imaging is critical, especially in children with persistent symptoms unresponsive to conventional treatment. In all 3 cases, imaging confirmed a right-dominant DAA forming a complete vascular ring, guiding surgical intervention.

Surgical repair is the primary treatment modality to relieve the vascular compression on the trachea and/or esophagus and is recommended for patients exhibiting symptoms of tracheal or esophageal compression, and a supplementary procedure in those undergoing corrective procedures for other cardiothoracic anomalies.^5^^,^^10^ Surgical repair is preferred to be performed soon after diagnosis, typically at a median age of 6 months, because even asymptomatic vascular rings often progress to significant airway symptoms, and early intervention can prevent serious complications such as hypoxia, dysphagia, tracheobronchial injury, or even sudden death associated with delayed treatment.^5^^,^^10^ Open surgical approaches are the most frequently reported and rely on the fourth intercostal space as an access point to the chest cavity, followed by lung retraction and pleural incision; the vascular ring is then exposed along with key surrounding structures including the esophagus, trachea, phrenic nerve, vagus nerve, and recurrent laryngeal nerve, which are carefully identified and preserved.^5^^,^^10^ The surgical approach is typically determined by the side of the nondominant arch, which is most often left-sided in nearly 71% of cases, making left lateral thoracotomy the preferred method, whereas median sternotomy is used less frequently.^5^ In our study, all 3 patients underwent left lateral thoracotomy. DAA repair can also be done via a minimally invasive video-assisted thoracoscopic surgical technique successfully for selected vascular rings.^11^

Several complications have been reported, with chylothorax being the most common, followed by left vocal cord paralysis due to recurrent vagal nerve paralysis and transient postoperative hypertension.^5^ Additionally, rare cases of aortoesophageal fistula have been described, typically presenting with massive gastrointestinal bleeding.^12^ Furthermore, due to the procedure's proximity to critical structures, complications such as esophageal perforation and pneumothorax may occur, in addition to the usual surgical risks of bleeding and infection.

In our study, all 3 patients underwent surgical repair via left lateral thoracotomy. All procedures were completed without intraoperative complications, and postoperative recovery was uneventful in every case. Patients were successfully extubated within 24 hours, oral feeding was resumed early, and no reintervention was required. The left thoracotomy approach was sufficient to achieve effective anatomic correction and complete symptomatic relief, reinforcing its role as the standard surgical strategy for DAA with a left-sided nondominant arch.

Postoperative outcomes are generally good; however, persistent stridor remains a common postoperative symptom, seen in 30% to 40% of patients, has been linked to younger age at repair and longer intensive care unit stays, and is often caused by underlying airway abnormalities such as tracheomalacia, tracheal stenosis, or both.^5^ The prognosis is generally excellent, with reoperation required in only a small fraction of cases; however, mortality has been reported in patients with chromosomal abnormalities, cardiac defects, or severe postoperative complications such as pneumonia-related respiratory failure.^5^ Similarly, in our case series, none of the patients required reoperation, and although mild respiratory symptoms such as stridor and noisy breathing persisted in some cases, they improved significantly compared with the preoperative stage. These findings are consistent with the gradual improvement commonly seen postoperatively, as the airway remodels and pressure from the vascular ring is relieved.

## Conclusions

DAA, although rare, should be suspected in infants and young children with persistent respiratory or feeding symptoms refractory to standard therapy. Early diagnosis with appropriate imaging prevents morbidity from prolonged airway or esophageal compression. Surgical repair via left thoracotomy with division of the nondominant arch is safe, effective, and associated with excellent outcomes.

## Funding Support and Author Disclosures

The authors have reported that they have no relationships relevant to the contents of this paper to disclose.

## Appendix

Video 1 Transthoracic Echocardiography Suprasternal short-axis view with color Doppler, showing the 2 limbs of the double aortic arch forming a complete ring around the tracheal central air column.
